# Genetics of disease severity in multiple sclerosis, Alzheimer’s disease, and Huntington’s disease: rejuvenating genome-wide association studies

**DOI:** 10.1007/s00415-017-8584-y

**Published:** 2017-08-07

**Authors:** James Hrastelj, Neil Robertson

**Affiliations:** Division of Psychological Medicine and Clinical Neuroscience, Department of Neurology, Cardiff University, University Hospital of Wales, Heath Park, Cardiff, CF14 4XN UK

## Introduction

Genome-wide association studies (GWAS) now available for many common neurological disorders have been highly informative, for example, identifying more than 110 replicated genetic variants associated with multiple sclerosis (MS). However, there has been relatively limited progress in identifying genetic modifiers of disease severity and clinical phenotypes. This has now become an area of active research, since it offers a substantial opportunity to detect both prognostic biomarkers and novel therapeutic targets. Classical GWAS is a simple comparison of genotypes between cases and controls, but measuring disease severity is more complex and leads to substantial variation in methodology. A fundamental limiting factor to date has been the lack of large cohorts with detailed long-term clinical data that allow powerful and meaningful correlations to be drawn. However, recent work in three neurological disorders has been able to successfully overcome these historical limitations and have identified genetic variants that correlate with clinical outcome.

This month’s journal club discusses four recent papers that have used different approaches to identify genetic modifiers of clinical severity and phenotypes in MS, Alzheimer’s disease (AD), and Huntington’s disease (HD). With these methodological advances comes a need for even larger cohorts of genotyped patients with detailed longitudinal phenotypic data across the neurological disease spectrum to refine, replicate, and expand these findings.

## Genetic modifiers of multiple sclerosis progression, severity, and onset

Between 30 and 50% of MS risk is genetic, but there has been limited progress in identifying genetic variants that affect disease severity or outcome. In this month’s first paper, Sadnovick et al. report results of an ‘extremes of outcome’ analysis of 2016 patients with MS to identify genetic variants that influence age at onset (AAO) and rate of disability accumulation. Fifty patients at each end of the disability spectrum underwent exome sequencing. This approach detected 38 missense or nonsense variants with nominally significant difference in frequency between the groups.

The 38 identified variants were genotyped in the remainder of the cohort to seek correlation with severity, clinical course, and AAO. The multiple sclerosis severity score (MSSS) was calculated for each patient from a single EDSS measurement and was used in a linear regression model adjusted for sex and AAO. Two variants were found to have nominal significance: one in the gene *PSMG4* and one in *NLRP5*. Variants in these two genes also correlated with earlier AAO, while a variant in *MC1R* appeared to delay onset by nearly 2 years. Clinical course was correlated with a variant in *EIF2AK1*, which was associated with a 50% greater risk of primary-progressive MS relative to relapsing-onset MS.

Of the genes identified, *PSMG4* and *MC1R* are perhaps the most interesting. *PSMG4* encodes a chaperone protein involved in assembling the 26S proteasome, a major protein disposal pathway. Reduced activity of the 26S proteasome has been shown to cause neuronal death by impaired degradation of abnormal proteins. The 26S proteasome has also been shown to hydrolyse myelin basic protein to produce antigenic peptides for presentation to T cells. *MC1R* encodes the receptor for melanocyte-stimulating hormone and the identified variant is associated with the phenotype of red hair, pale skin, and freckles. This phenotype facilitates synthesis of vitamin D in low UV light environments and so may confer protection through vitamin D-related mechanisms.

*Comment*. Exome sequencing in patients at the extremes of outcome maximises power to discover high impact variants. Several genes were identified, but none survived experiment-wide correction for multiple comparisons and there was no adjustment for treatment, so replication will be imperative before firm conclusions can be drawn. Exome sequencing restricts analysis to the coding genome, so future work should extend to the non-coding genome, because genetic modifiers of MS severity may lie in regulatory regions. Finally, using the MSSS as a severity measure is problematic for several reasons. First, it has not been validated longitudinally against long-term disability data and so may not be a stable measure of long-term outcome. Second, it relies on a single EDSS measurement, which is highly operator-dependent and prone to inaccuracy. Third, the distribution of MSSS data is highly variable, so linear regression may not be applicable. Future work should consider survival analysis of long-term disability data to produce reliable, sensitive, and clinically meaningful results.

Sadnovick AD et al (2017) Clin Immunol 180:100–105.

## Gene variants of adhesion molecules act as modifiers of disease severity in MS

A second method to identify novel treatment targets is to restrict analysis to genes involved in modifiable pathways. Dardiotis et al. genotyped 147 single nucleotide polymorphisms (SNPs) in nine genes involved in trafficking of lymphocytes into the central nervous system (CNS) in 389 Greek patients with MS. They performed linear regression for AAO and MSSS, adjusting for sex and study site in the former and treatment (cumulative duration) and AAO in the latter, with significance determined by permutation. One variant in gene *FN1* was found to associate with AAO and two variants were found to associate with MSSS: one in gene *ITGA4* and one in gene *SPP1*, with each SNP exhibiting an allelic dosing effect.

*Comment.* While the sensitivity of this work may have been limited by the deficiencies of the MSSS, this paper suggests that mechanisms regulating lymphocyte trafficking into the CNS are important in determining MS severity. Targeted analysis of selected genes with known functions can yield important information about disease mechanisms. One of the genes identified in this study is the molecular target of natalizumab, a potent disease-modifying drug for MS, illustrating the potential of such an approach for identifying novel therapeutics.

Dardiotis E et al (2017) Neurol Neuroimmunol Neuroinflam 4(4):e350.

## Identification of genetic variants associated with Huntington’s disease progression: a genome-wide association study

Monogenic disorders, such as HD, provide a powerful model for detection of genetic modifiers of disease outcomes. In this study, Moss et al. show how highly detailed longitudinal clinical data from a modest cohort can lead to successful detection of genetic modifiers of disease severity on a genome-wide scale.

TRACK-HD is a prospective observational study of 216 patients with HD with highly detailed clinical and brain volume MRI data over 3 years. Progression slopes for outcome measures that were correlated with each other and independent of CAG repeat length and age were included in a principal component analysis to produce a TRACK-HD progression score. Genome-wide chip-based SNP genotyping was performed and a mixed linear regression model applied, treating TRACK-HD progression score as a quantitative trait. A locus on chromosome 5 was identified that reached genome-wide significance, which spanned three genes: *DHFR*, *MSH3,* and *MTRNR2L2*. The finding was replicated by analysis 1773 individuals from REGISTRY, a multicentre, prospective, observational study of patients with HD. Despite REGISTRY data having fewer measurements, significant missing data, and no imaging, genome-wide association analysis of the first principal component of the most recent data identified the same locus on chromosome 5.

The index SNP is a coding missense variant in *MSH*3. *MSH3* encodes a neuronally expressed DNA mismatch repair protein, which has been implicated in HD pathology in mouse and in vitro studies. DNA repair pathways were also significantly associated with progression and gene expression analysis showed that reduced expression of *MSH3* was associated with slower disease progression.

*Comment.* This paper reports a genetic locus that appears to modify progression in HD. The findings support the model that somatic expansion of the CAG repeat in *HTT* is a driver of HD progression. The work also shows the utility of detailed phenotypic data to reveal genetic modifiers of severity on a genome-wide scale. Interestingly, despite larger numbers, the association was weaker in the REGISTRY, the cohort with patchy clinical data. Combining detailed clinical data across multiple modalities may produce important insights in other neurological disorders.

Moss DJH et al (2017) Lancet Neurol.

## Genome-wide association study identifies four novel loci associated with Alzheimer’s endophenotypes and disease modifiers

The final paper discussed that this month illustrates the power of using endophenotypes to identify genetic associations with disease risk and severity. Endophenotypes are measurable traits that strongly associate with a disorder and share its genetic architecture. Endophenotypes can, therefore, be used as quantitative surrogate markers of disease. This allows greater sensitivity for detecting genetic risk variants in smaller sample sizes, but also provides mechanistic information linking genotype to disease. Deming et al. performed extensive analysis of 3146 patients with AD from nine studies, utilising well-established endophenotypes of AD: cerebrospinal fluid (CSF) levels of amyloid-β (Aβ), tau, and phosphorylated tau (ptau).

All patients underwent chip-based SNP genotyping. After normalising CSF measurements across different sites, a combined GWAS was performed applying linear regression of each SNP on CSF Aβ, tau, and ptau levels, adjusted for study, age, and sex. In addition to replicating previously associated loci, they identified two novel associations with ptau and Aβ: a variant on chromosome 13 near *PCDH8* and one on chromosome 18 near *CTDP1*. Aβ was also associated with two further novel variants: one on chromosome 1 near *GLIS1* and one on chromosome 6 within *SERPINB1*. Further analysis suggested that approximately 35.5 and 24.9% of the variance in CSF levels of Aβ and ptau, respectively, were explained by common genetic variants.

The authors then investigated the genetic overlap between variants associated with CSF Aβ, tau and ptau levels, and AD risk, age at onset and clinical progression rate in independent cohorts. Progression was measured by the annual change in Clinical Dementia Rating Sum of Boxes (CDR-SB) over at least 1.5 years following diagnosis after adjusting for age, sex, baseline CDR-SB, follow-up time, level of education, and site. The novel variants associated with Aβ (*GLIS1* and *SERPINB1*) were also associated with AD risk, AAO and progression, and previously reported variants associated with tau and ptau also associated with AD risk and AAO.

*Comment.* This is an extensive paper, but the key findings establish genetic variants that associate with CSF levels of Aβ, tau, and ptau, which also correlate with AD risk, AAO, and rate of progression. This reinforces the potential of these CSF proteins as biomarkers of prognosis, as well as diagnosis and illustrates how endophenotypes can be used to identify informative genes despite modest sample size.

Deming Y et al (2017) Acta Neuropathol 133(5):839–856.

